# Supplementary figures and images for: Shell density of planktonic foraminifera and pteropod species Limacina helicina in the Barents Sea: Relation to ontogeny and water chemistry
Source: PLoS One. 2021 Apr 28;16(4):e0249178. doi: 10.1371/journal.pone.0249178 (PMC8081242; doi:10.1371/journal.pone.0249178)

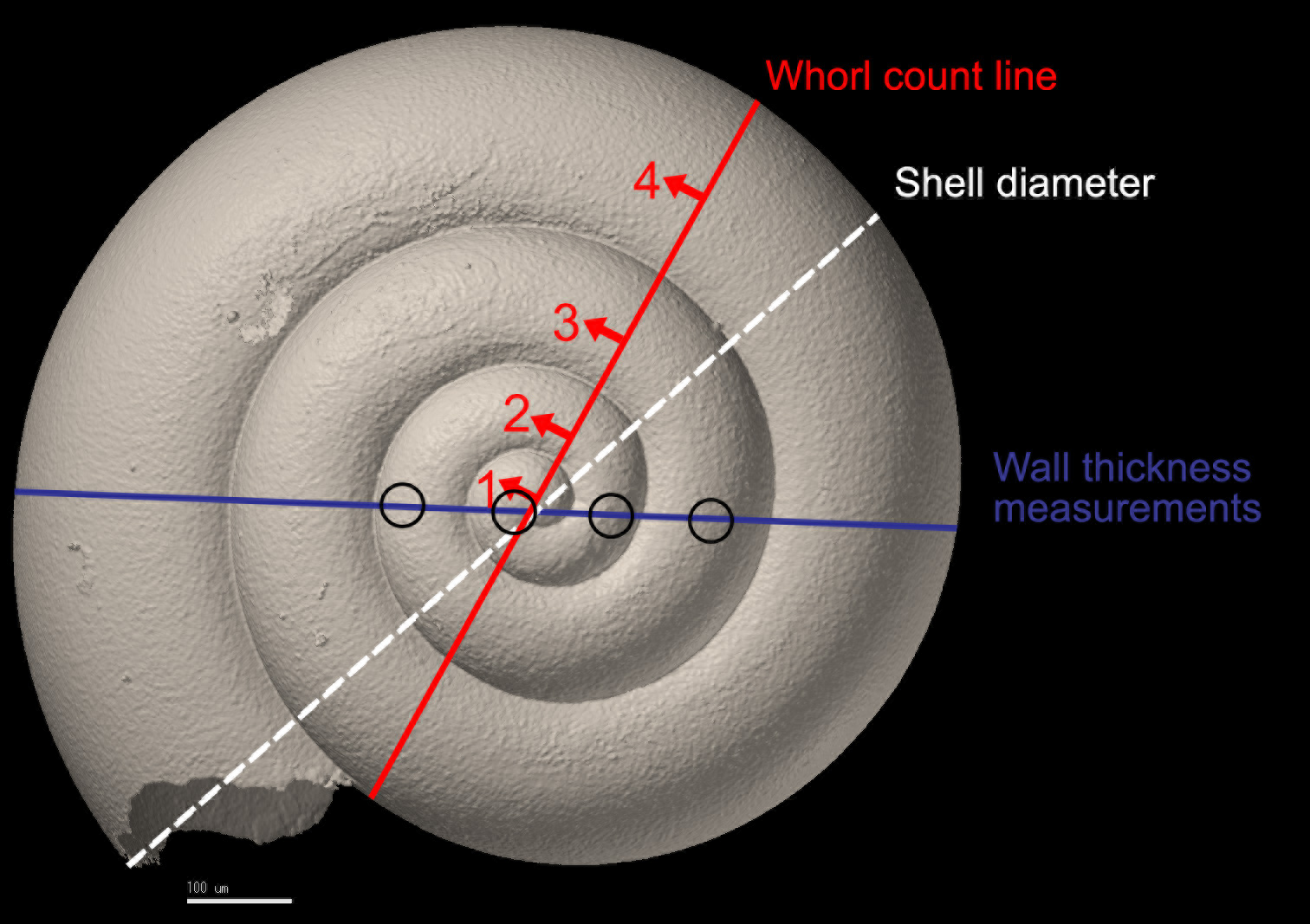

Supplement: S1 Fig — Wall thickness measurements were done along a cross-section (blue), and diameter measured along white stippled line. Black circles show location of shell thickness measurements. The shell in the figure has 3.5 whorls. More details on whorl counting method can be found in Janssen [53]. (TIF) [file pone.0249178.s001.tif]

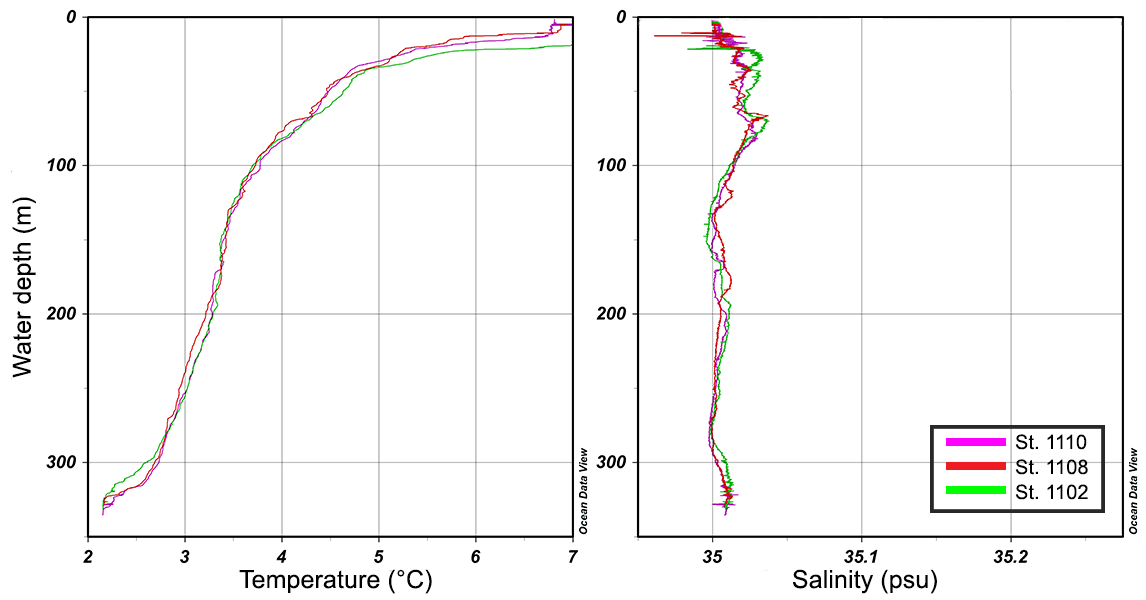

Supplement: S2 Fig — (TIF) [file pone.0249178.s002.tif]

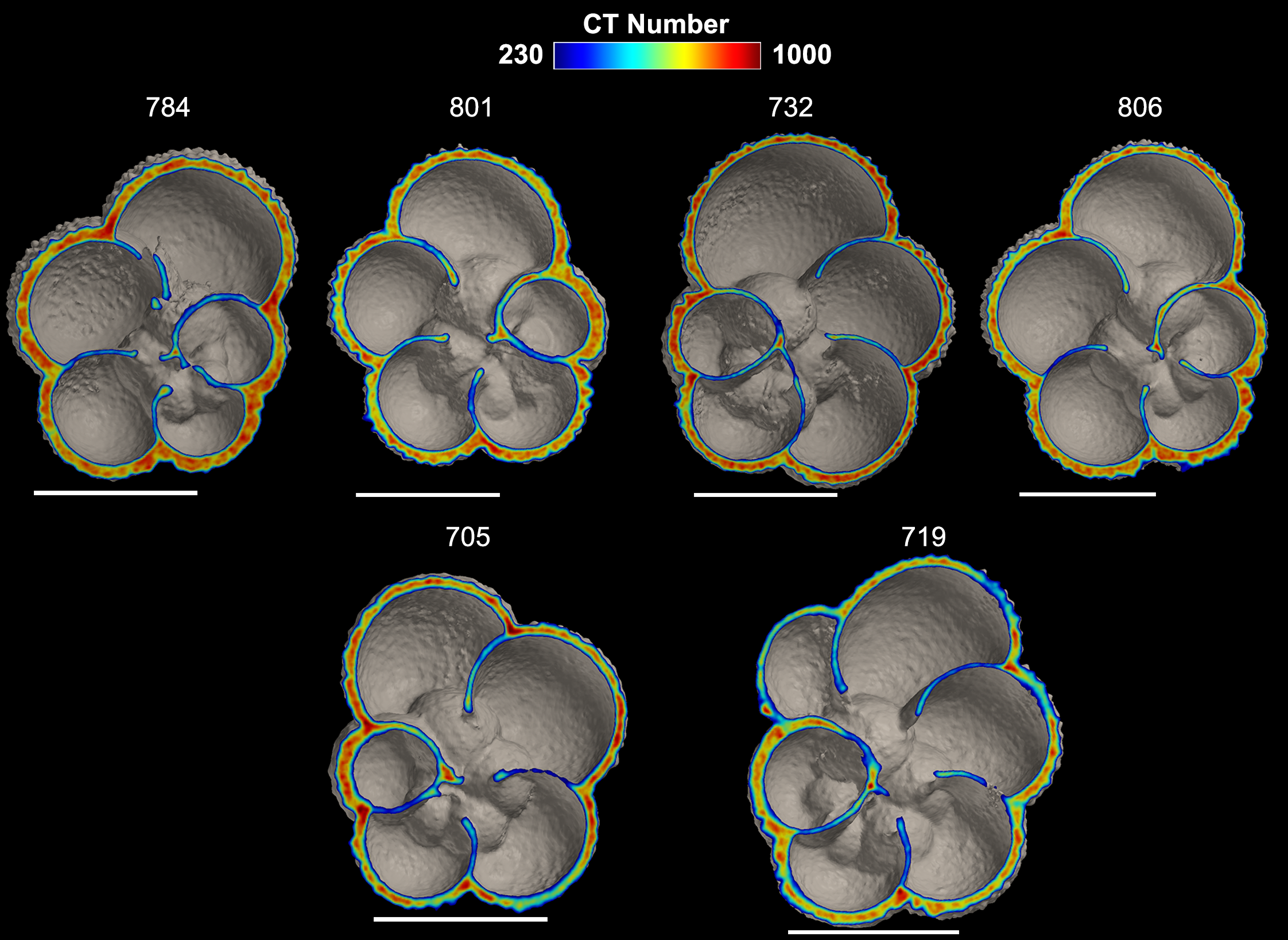

Supplement: S3 Fig — Scale bars measure 100 μm. (TIF) [file pone.0249178.s003.tif]
