# Supplementary material for: Shell density of planktonic foraminifera and pteropod species Limacina helicina in the Barents Sea: Relation to ontogeny and water chemistry
Source: PLoS One. 2021 Apr 28;16(4):e0249178. doi: 10.1371/journal.pone.0249178 (PMC8081242; doi:10.1371/journal.pone.0249178)
Supplement: Attachment — Submitted filename: Review of Relationship between shell density and vertical distribution of living planktonic foraminifera and pteropod species Limacina helicina in the Barents Sea.pdf [file pone.0249178.s005.pdf]

Review of Relationship between shell density and vertical distribution of living planktonic foraminifera and pteropod species *Limacina helicina* in the Barents Sea

This manuscript presents CT scans of planktonic foraminifera and pteropods from the Barents Sea. The title suggests that the study is focussed on morphological changes, namely shell density, through a vertical profile of the water column, however, the study site is only 340m water depth with strong lateral currents from the North Atlantic. It is not intuitive to me why the authors would have selected this location if the title were to reflect the original objective of this study.

There is no real story in this data. The data is poorly represented, in fact I'd go as far as to say misleadingly so, in order to derive any story at all. The story that is drawn, that pteropods adapt to ocean acidification by thickening their shell, is entirely unsubstantiated by the data presented here.

I summarise some of my issues below.

Introduction. I found the introduction to be poorly written and often ambiguous, leaving the reader guessing at what is meant… e.g. what is meant by "positive relationship" between ocean acidification and food? Moreover, the authors display no critical assessment of previous research. I would have liked the authors to show that they had considered the limitations or caveats of past studies, especially an appreciation for the limitations of experimental/incubation studies verses field observations, rather than implying that all studies are equally robust. Ocean acidification studies are full of inconsistencies and opposed methodologies. We can all learn from what has and has not worked in the past, it is a real shame that the authors did not take this opportunity to say why their study would make an advancement.

Method. For the foraminifera it is very clear that the orientation of the cross section, and how much of the inner shell walls are sampled, has an influence on the mean CT number. I suggest that this analysis could be more robust had the CT number only taken into consideration the shell wall of the penultimate chamber. i.e. inner shell walls may be reabsorbed during the forams life cycle and/or disproportionally sampled by the cross section. The final shell wall may still be growing. The penultimate shell wall, and only that part that has an outer surface, could arguably be a more consistent/robust measure than the mean of the entire cross section.

Results. I took the time to look at the excel file of data supplied as suppplement. I was unable to reconcile the values in the "estimate" columns in tables S4 and S5 with the raw data. What is "estimate"? Furthermore, the units in these tables do not make any sense… I am pretty sure that the shell thickness is not >40000 mm! I am not an expert in stats, but I know enough to understand that the stats do not really support the statement on line 280 or any of the following conclusions. The r2 values are nothing to write home about. I suspect that had a deeper water site been chosen, there may be more of a story in the vertical profile variability.

Line 285. It is revealed that up to 85 % of specimens recovered from >150m water depth were "not living". There is no mention of these specimens being dealt with differently/isolated from statistical analysis. Rather, in line 291 and again in the discussion, statements like "gradual shell thickening with depth" with no further recognition of the fact that many/most specimens are dead and therefore their position in the water column is more likely to reflect settling out/reworking by lateral/upwelling currents and/or "reworking" by predators. Once you've revealed that the specimens are not living it becomes a nonsense to draw conclusions are the life stages/depth migration of these specimens.

Line 292+ Foram shell thickness of 2-4 microns? I suspect that is an order of magnitude out. Had the authors looked at other seminal works on N. pachyerma sinistral they would have known that there measurement of shell thickness was way out. Kohfeld and Kozdon references are amongst critical references omitted from this manuscript.

Line 295. What is μm^{-m}?

Line 300. This is the ONLY parameter compared with shell thickness!

Line 324. What is this 2-layer dissolution pattern? I don't see it in the figure.

Line 355. Because Ω varies with depth!

Line 357. The authors conclude that shell thickness of L. helicina increases in sequential whorls. This is absolutely not supported by the CT images. i.e. the thickness of the shell whirls shown in figure 8f do not support the plot shown in 8d. The only part of the shell that shows anything like the thickening reported in the plots is the structural part of the shell at the central spiral which must increase with each whorl for structural reasons. There is absolutely no evidence to support thickening of the shell as an adaptation to ocean acidification. This is a poor attempt to drag a story out of this data. The discussion from line 483 onwards, based on these ill found conclusions, is completely unsubstantiated.
