# Supplementary material for: Shell density of planktonic foraminifera and pteropod species Limacina helicina in the Barents Sea: Relation to ontogeny and water chemistry
Source: PLoS One. 2021 Apr 28;16(4):e0249178. doi: 10.1371/journal.pone.0249178 (PMC8081242; doi:10.1371/journal.pone.0249178)
Supplement: Attachment — Submitted filename: Review_for_Ofstad_et_al_2020b.pdf [file pone.0249178.s006.pdf]

Review of Ofstad et al. for PLOS One

B. Metcalfe

The authors have used water column measurements of carbonate parameters in conjunction with XMCT to determine the thickness of shells of *N. pachyderma*, *T. quinqueloba* and the pteropod L. helicina from plankton tow data from the Barents Sea. It is well written and an interesting paper with a lot of new data. There is a large amount of speculation (e.g., lines 511) within the discussion section which I don't dislike (there are some interesting ideas) but I would suggest that the authors more clearly frame it in 'the requirement for clear biomonitoring to establish whether such hypotheses are true or not' this would also fit with their own abstract (i.e., line 46). There are some discrepancies (though minor) within the text and the supplement (see comments below), I suggest major revisions to give the authors time to double check the supplement that was uploaded and clarify parts of their methodology.

Answers to PLOS One's questions

1. Is the manuscript technically sound, and do the data support the conclusions?

Yes

2. Has the statistical analysis been performed appropriately and rigorously?

Yes. However, please include the statistics that relate to the following statement (line 42-44:' We show that the density of L. helicina shell has an inverse relationship with ΩAr and hypothesize that the gradual thickening of the shell wall could be an adaptation against low Ω')

3. Have the authors made all data underlying the findings in their manuscript fully available?

Yes. However, the authors should include in the supplement the net min, max, mid depth intervals environmental parameters (e.g., temperture, salinity, omega, etc) alongside what they have already included.

4. Is the manuscript presented in an intelligible fashion and written in standard English?

Yes

Major comments

There is some missing information from the methodology which should be elaborated upon in this paper. For instance the authors have weight (supplementary table 4 and 5) but no mention of how it was measured/calculated (what device, in groups, individually?). Were the foraminifera picked from >63 um? From a recent publication the authors picked from 150-200 um, are these the same specimens or were the specimens for isotopes picked first? Etc. etc. Please expand the methods section.

From the mean CT number you can have variation either from the total number of voxels with a specific ct number or with voxels from the whole test. I.e., the ct number can apparently reflect size change or thickness/volume change. The authors do point out that size variation can disrupt their data ("outliers

may occur if specimens have an unusual morphology, as for example in the T. quinqueloba plot, there is an individual with an abnormally large final chamber, which also has a low density (Fig 5B)”). Have you considered further normalising each ct number by the size of the shell to remove the potential change in size with depth complicating your results? I do wonder if this already done in the ‘thickness’ measurement which is volume/surface area, which to me sounds more like the volume of calcite normalised to size rather than a measure of the thickness of the shell.

Line 42-44 ‘We show that the density of L. helicina shell has an inverse relationship with ΩAr and hypothesize that the gradual thickening of the shell wall could be an adaptation against low ΩA’. I don’t actually see where this is directly compared (apart from plotting in Figure 8 a panel with the ΩA values next to a panel with CT number), there is a comparison with depth (supplement table 6) but no direct comparison (r2, p value) between ΩA and CT number. Please add this into the results section (line 355). Furthermore, in the supplement I don’t see any tests against environmental variables, whilst most environmental parameters covary and most changes can be seen with depth, the authors have chosen only to test depth. Whilst culture studies have shown a relationship with carbonate parameters, from your data you could also argue that shell density has an inverse relationship with temperature.

Out of curiosity what is the rationale for plotting the maximum depth of the net interval as opposed to the mid-point? As depth is an explanatory variable and tested rather than environmental parameters why choose the deepest depth? How does shifting the depth to the minimum or midpoint impact your r2.

In section 3.1 you identify two distinct layers, could you not test statistically the difference between shells in the upper layer and the lower layer. Normalising for size change to see if they are differences which are statistically significant.

Whilst it’s not a major comment, the use of LPF instead of living planktonic foraminifera seems to be an unnecessary acronym e.g., line 473 ‘This scenario may be found when LPF calcify in low…’ do you need ‘living’ in this sentence? It would be precipitation rather than calcification if they were dead. Or line 524-526 ‘living planktonic foraminiferal (LPF) species Neogloboquadrina pachyderma and Turborotalita quinqueloba and the living pteropod species’ extant or modern would be the preferred terms.

Finally, I have tried to note down lines in which the references need to be adjusted to fit PLOS’s reference style, these are: 65-66; 90-91; 151; 154; 166, 212, 578.

Minor comments

Line 60: ‘Also, the cold surface water has greater CO2 solubility’. Personally I would put a rephrased version of this (e.g., ‘The solubility of CO2 in seawater has a relationship with temperature’) at line 57 just after ‘ocean acidification (OA).’

Line 61 remove 'that', replace 'is' with being : "results in the Arctic Ocean being particularly"

Line 62-66: clarify for the reader whether this is only surface waters or includes intermediate / deep (give the potential depth habitat of foraminifera, pteropods etc)

Line 68 'and cause' replace with 'with an associated'

Line 69: 'the observed ocean' – are you still talking about the Barents Sea here or the Arctic Ocean?

Lines 66-73: You can condense this section, e.g., 'The Barents Sea is one of the largest CO2 sink areas in the Arctic region, which is 67 mainly caused by the year-round CO2 undersaturation and high biological production [13,14] **despite the formation of sea-ice in winter.** The Barents Sea CO2 sink is predicted to double by 2065 with an associated a pH decrease of up to 0.25 pH units [15]. **A significant proportion** of the observed ocean CO2 increase between 1967 and 2001 of 42 µatm **has been** from the inflow of Atlantic water, which is rich in anthropogenic CO2 [16]. The meltwater from sea ice or glaciers lowers the ΩAr and ΩCa [17–19], and is predicted to increase as a result of warming [20].'

Line 83 define LPF ('There is no known ΩCa thresholds for *LPF*') rather than in lines 163 or 393 and is this sentence a repeat of the line 85 ('There **is** no known ΩCa thresholds for planktonic foraminifera')? Regardless the word 'is' should be 'are' as you are not talking about a singular thing.

Line 91-93: I would modify the rationale a bit (i.e. directly answer why it's necessary to have in situ studies if culture studies have shown X – this also links back to your biomonitoring in your abstract) : 'Having demonstrated the impact of lower CaCO3 saturation states in culture, it is important to obtain in situ data for comparison and to put culture experiments into context through attempts to link species biological response to changes in ocean chemistry.

Line 98 replace 'but this' with 'a similar'. Or remove this section and add it to the end of line 101.

Line 105: 'There is a lack of established methodology to study biological effects of OA.' The subsequent discussion is with respect to 'calcification' perhaps alter this sentence to reflect this?

Line 110 'have become **better** refined and accurate over time'

Line 163: I don't see the necessity in using LPF if it reflects both empty and cytoplasm bearing shells (i.e., it would be better to use when only referring to cytoplasm bearing foraminifera)

Line 166: I would include all pertinent procedures and chemicals i.e., from the reference given, give a brief version of the plankton tow and sediment procedures. I.e., repeat and rephase both " Immediately after recovering the sediment core, the top layer (1 cm) was scraped off by a spoon, and preserved in approximately 50 ml of ethanol (96%) with rose bengal (2 g L^{-1} of ethanol), and stored at 2 °C."

… and "the samples were sieved with sea water through a 63-μm sieve and transferred into plastic bottles (250 ml) where they were immediately fixed and buffered with approximately 230 ml ethanol (98%), a quarter of a teaspoon hexamethylenetetramine (≥99.0%), and stored at 2 °C. Once returned to the home laboratory, the samples were briefly washed with tap water on a 63-μm sieve in order to remove organic particles from the surface of the foraminiferal tests and to break up aggregations of material."

Line 166: I would include some information of how you selected the specimens. Were all foraminifera picked from >63um? Were the shells picked 'randomly' or a selection of specimens ranging in size? In Ofstad et al., (2020) the foraminifera were picked from 150-200 um is this the case here?

Line 184-186: Dividing the volume by the surface area to calculate the thickness, doesn't this massively over simplify the thickness? To me it sounds more like the volume normalised to shell size than 'thickness' of the shell. How is the surface area chosen of a 3D object, is it the maximum surface area?

Line 191: "Live (cytoplasm bearing) planktonic foraminifera specimens were counted for each depth" seems out of place here in the XMCT section, perhaps move to prior section/ line 166

Line 211: 'nearest 10$^{th'}$, does this mean that your whorl measurements are rounded to ~18 degrees (half of 36)? Include Janssen's (2007) point that the determination of "estimating the ultimate whorl" has "an accuracy of a quarter whorl".

Line 215: have you determined what is the error associated with the adobe ruler tool? E.g., repeat measurements of the same shell wall? From experience with Photoshop ruler, ImageJ and all manner of other measurement software I have determined thickness, shell size, etc from a couple of measurements just to average out potential undulations in wall, etc.

Line 256: missing '–' between 200 and 300 m

Line 247-256 / Line 270-277: The authors have brought up that their increase should be viewed in light of the range in depth ("Although we found a general increase in CT number and shell thickness with

depth, we note a large range in CT numbers (Fig 2 and S2 Table) and mean shell thickness (S2 Table) at each sampling depth interval.”), I agree with them, have they considered testing whether the difference between depths is statistically significant? Rather than testing the significance of the linear fit, testing whether the samples within each net are statistically different from one another. Plotting the min, max, mean with std dev as error bars from table s2 it does seem apparent that there is a greater shift in the minimum than the maximum for the ct number.

OR plot the raw data as four panels in boxplots for figure 2 (see figure comments below)

Line 280: “Both *N. pachyderma* and *T. quinqueloba* show a statistically significant positive correlation between individual shell weight, CT number, mean shell thickness and area density with water depth (S4–S5 Table)”. Where are the intercepts in the supplementary table? The ‘estimate’ is the slope, no?

Line 346: In your supplementary table (limacine helicina tab) I am confused about how the numbers of whorls corresponds with measurements labelled whorl 1, whorl 2, whorl 3, whorl 4. For instance how can a shell with 0.6 whorls have measurements in whorl 1 and whorl 2 column? Especially if the shell in fig s3 has 3.5 whorls and has four measurements. The same for 1.3 whorls having measurements in whorl 1, 2, and 3 columns (there is also a discrepancy some 1.2 whorl shells have 3 measurements some have 2). I.e., how does a shell have measurements for whorls it does not have (or have I read this wrong?).

Line 356: A further question, looking at the supplementary table, is how comparable is a whorl measurement made on X.3 of a whorl compared with say X.8. In figure S3 your stars seem to avoid the shell opening (understandably given the difficulty in obtaining a reliable measurement there), but I assume with a smaller amount of whorl grown the measurement of shell thickness has to be made closer to the opening of the shell. Have you tried to estimate the impact of this on your measurements? Is some of the variation between the same whorl number but for different shells partly to do with how close the measurement is made to the opening of the shell?

Line 349: In your supplementary table there doesn’t appear to be a shell with 3.1 whorls, at 200-300m (the only corresponding values are those made on shell 1108 200 pt14 with 2.8 whorls), and the measurements of the zoomed in section for the 1.8 whorl shell comes from a shell with 2 whorls in the supplement.

Line 351: indicate in the caption what type of ‘error bar’ the lines on the bar chart refer to (standard deviation, standard error, CI?). And what are you plotting here, the mean?

Line 361: ‘while shells with less or equal’ , your supplementary table uses ‘equal less than 2’ (with data = 0.6-2.0) and ‘greater than’ (with data = 2.0-3.6). Check if you mean 2 or 2.3 and check whether shell ‘1108 100 pt3’ is missing a decimal place (otherwise it should be in the equal or less). I would reorder the supplement so that it corresponds with the value quoted in the text . Furthermore, as you have

data for the top measurements only from shells with 1.5 -2.3 and 2.3-3.6 whorls, would it not be better to word it as such?

Line 359-362: Could you explain the rationale for using the sum of the four values as opposed to say the arithmetic mean? Likewise what is the rationale for splitting the measurements into < 2.3 whorls and >2.3 whorls? Was this related to the number of samples or does it have a biological/morphological rationale? Whilst the authors should check (see previous comment) if they mean 2? What would happen if you split the dataset in a different way.

Line 360-362: "The sum of four measurements done on the central-top part of the shell show that…"

(replace done with made)– Are some of these four measurements from the same whorl? Its hard to tell with a 3D image, perhaps indicate on figure S3 so that a reader can see how the stared thickness measurements correspond with these top measurements. In the limacina helicina supporting information why do some have Top 7; Top 6; Top 5? What do these additional measurements correspond with?

Line 400: The North Pacific data of *G. bulloides* uses area density and outer wall thickness, so the percentage change might not be equivalent. Have you considered computing the same methodology as ref 31 (Iwasaki et al., 2019), (1) to see if the methodologies are comparable and (2) to directly compare the values here.

Line 405-408: Could the changes also reflect the fact that the species is spinose (http://www.mikrotax.org/pforams/index.php?id=104250). So differences between non-spinose (N. pachyderma) could reflect different calcification mechanisms

Line 409-412: 'If we use thick density shells as a proxy for reproduction' Why not use the appearance of non-cytoplasm bearing shells as a proxy?

Line 417: some early culture papers suggested that wall dissolution could be a common occurrence

Line 420-424: What about the sinking of dead shells, if as Takahashi and Bé suggested in their sinking speed studies sinking speed is related to shell size/weight. Then the decrease in density at the deepest nets could also reflect a relic population (or some specimens of such a population) that haven't sunk far enough yet.

Line 447: replace fragments with fragmentation

Line 454 - 461: “The steeper *N. pachyderma* curve suggests that they continue to calcify for longer than T. quinqueloba” whilst its true they seem to have calcified more it’s not possible to extract a rate or length of time. I would avoid extrapolating too much from your dataset, your data comes from several hours so its next to impossible to really test the arguments made here. The same data could be used to argue a longer life cycle just as much as it could be used to suggest that the species calcifies more often; produce more calcite per calcification event (maybe spinose species invest more time building spines), etc.

Line 470: ‘shells which have undergone either post-depositional dissolution or calcified in low ΩCa waters’ – include reference 46 (Johnstone et al., 2010) here as this is an idea very similar to what they proposed

Line 473-474: “This scenario may be found when LPF calcify in low ΩCa waters, and shift their ecological strategy to favor shell size over shell density [31].” – Although *T. quinqueloba* is known to have a quirky shell outline with considerable variability of form related to multiple environmental parameters (if you can get access to it, see ‘Phenotypic variation of Turborotalita quinqueloba (Natland) tests in living populations and in the Pleistocene of an eastern Mediterranean piston core’, D Kroon, PF Wouters, L Moodley, G Ganssen…et al).

Line 493: ‘and as long as the ΩAr is sufficiently high’ – you could replace sufficiently high (is 1.2 high?) with ‘and as long as ΩAr is sufficient (>1.2)’

Line 501-510 and 511-522: Some of the discussion points raised in these sections go beyond the specific study and would necessitate a longer targeted study (e.g., either long term plankton tows or sediment trap studies) to answer them all (e.g., “The sampled L. helcina population at the crater <u>area most likely were spawned</u>”; “ <u>they will prioritize</u> increasing their thickness and density in preparation for fall/winter when they most likely will experience a minimum in ΩAr”; “<u>We speculate</u>, that the decrease in ΩAr may lead to veligers”; “the veligers <u>may start</u> producing thicker and denser shells relative to what we see today”). Therefore, this would be a good point to bring in one of the conclusions raised in the abstract, lines 45-47 (‘This study clearly demonstrates the importance of biomonitoring to understand the variability in shell density of calcifying zooplankton’) that has so far not been directly raised. Perhaps rephrase the start along the lines of ‘A longer study using the techniques described here could answer….’

Line 524-525: living is redundant here, extant or modern etc. Do you mean specimens rather than species here?

Line 565 ‘sampling’ is missing a p

Line 570 remove ‘on’

Figure comments

Figure 2: The N. pachyderma data point at 200 m that is below or around 600 in CT number, this datapoint is lower than the value in table S2 for this species. Please check this datapoint. You have a minimum value for 484 for T. quinqueloba but your x-axis starts at 500. I would double check if merging the three plots hasn't led some datapoints to shift.

Figure 2: A lot of the visual impact of your data is lost by plotting all of the data together, why not plot as four panels (i.e., single panel for each species and a final fourth panel for the combined plots). By splitting the species specific data you could also indicate which specimen came from which plankton tow. A boxplot for this data (you can plot the raw data as a transparent overlay) would also give the reader an indication of the structure of the data (i.e., quantiles, median, etc). At the very least it is important to plot the min, max, mean, std dev so that readers can see the data in a clearer way.

Figure 3/Figure 4 Panel C: Plot the standard deviation of each mean. It is a nice visual plot, but I wonder why is the calcite saturation and depth 'undulating' , i.e., follow the lines. If there is suppose to be distance I would plot it. Furthermore, I understand 3 tows were combined but your plotting the mean of each depth vs. the variability in saturation along a transect. That does limit the comparison (we don't know which shells came from which tow), especially for depth interval 50-100m where there seems to be the 'largest' variation.

Figure 8D – I think you loose information by plotting it as a bar chart rather than as a box plot like E. The bars are simple so you can scale it down to fit with your figure so I understand why, but my problem is you can only compare the mean (? point out what the bars correspond to and what the error bars represent in the figure caption) all the information is lost about the lower end of each distribution (e.g., are there shells whose 4th whorl have thicknesses similar to their 1st whorl?)

Fig. S1: Add in the temperature and salinity datapoint for 300 m. You've plotted the mid points of the first and fifth net interval, I would do the same for the other intervals. This goes for Table S1 and S2, you have only given a temperature without indicating whether it's the minimum or maximum depth. I would include the environmental parameters at min, mid and max depth intervals so that future researchers could incorporate your work into a meta-analysis.

Table S1 for station 1110 are the sample numbers correct? You have mean calcite volume data for nets with 0 samples (I am guessing that column E, F, G may not be correct)

Table (limacine helicina tab in supplement): rearrange rows 31-58 (i.e, the samples with less than or equal and greater than) so they correspond to the value of 2.3 in the text. Explain what Top 5 to 7 correspond with.

Supplementary table. It’s a bit of work, but it might be prudent to include a readme section to explain some of the variables in your table(s).
