# Supplementary material for: Shell density of planktonic foraminifera and pteropod species Limacina helicina in the Barents Sea: Relation to ontogeny and water chemistry
Source: PLoS One. 2021 Apr 28;16(4):e0249178. doi: 10.1371/journal.pone.0249178 (PMC8081242; doi:10.1371/journal.pone.0249178)
Supplement: Attachment — Submitted filename: Comments for manuscript.pdf [file pone.0249178.s008.pdf]

Comments for "Relationship between shell density and vertical distribution of living planktonic foraminifera and pteropod species *Limacina helicina* in the Barents Sea"

I'm glad to have an opportunity to read a new manuscript to assess the shell density calcareous organism by MXCT scanning. It has been considered that the calcification of calcareous organism will be suffered from ocean acidification in the future, and number of studies tried to figure out the effect of ambient seawater acidification to their calcification intensity in the nature and culture experiment. In this study, authors focused on the two planktic foraminiferal species (*T. quinqueloba* and *N. pachyderma*) and a species of pteropoda (*L. helicina*) obtained from arctic sea, where there is concern about the ocean acidification progress. Using by sample set of plankton tow, they investigated the vertical distribution of shell density and its variance with migration under the present nature condition. Such basic information of variation in shell density with normal ontogenetic process is valuable for understanding the alternation of shell calcification condition due to external factor as like as ocean acidification in the following step of study. The research methodology and quality of data are fine, and I basically agree with their discussion. I believe that this manuscript is definitely suit for publishing from PLOS ONE. However, in order to make this manuscript clearer and easier to understand for reader, I'd like to suggest some points as follow.

Major comments

1. First, authors discussed about the vertical distribution of shell physical condition of living calcareous organisms against the background of the issue of ocean acidification. However, what they have done is the observation under the single environmental condition. Therefore, this study reveals the growth process or ordinary life cycle of calcareous organisms that is ontogenetic process in other word. On the other hand, they did not compare the shell physical feature under the different acidification condition at different place or time. This means that it is difficult to discuss the impact of ocean acidification to calcification intensity of calcareous organisms from the sample set in this study. Therefore, I am afraid that the discussion concerning the effect from ocean acidification, in particular pteropoda *L. helicina*, may be going beyond the scope of this study. In order to make this manuscript simpler and clearer, I suppose that authors should clearly explain the purpose and significance of this study in the Introduction. I know that this study uses valuable samples in the Arctic sea, focus on the main calcareous organism, and analyze shell density, which is closely related to ocean acidification. I highly evaluate these points and understand the significance of this study that against the background of ocean acidification. However, I suppose that author should clearly explain the importance for investigating the

ontogenetic and growth process of these organisms. In the current manuscript, there seems to be logical gap between introduction and three research purposes. By reconstructing the Introduction with this in mind, I think it will be much easier for reader to understand well.

2. Second, I have something to worry about the shell samples from the surface sediments. In this study, authors also discuss about the shell samples from the surface sediment samples. However, I feel that this discussion is different from main theme of this study. I could not understand well why authors had to measure the shells in sediment sample. If they are definitely necessary in this study, authors should explain why it is necessary and what is the purpose of it. Furthermore, because some basic information of sediment samples is lack from this manuscript and figures, it is difficult to understand. The detail of samples can be quoted from other papers, but at least sediment samples location, water depth and super/under saturated to carbonate should be represented in the manuscript and/or figure of Map.

3. Finally, the description of Results is difficult to follow for me, because there are frequent back and forth of figures and tables. Authors should reorganize the description of Results. The vertical profiles of shell density shown in Fig. 2 are also shown in Fig. 3 and 4. I suppose that it might be better to summarize in one figure or divide into the figures of each species. In addition, much information is shown in Fig. 3 and 4, but I suggest to add some words to explain the origin of sample or remarkable points in these figures. I hope it makes this manuscript clearer.

Other comments

Introduction

It is important to review the effect of ocean acidification to these calcareous species as already described in the current manuscript, but it is also important to mention the ontogenetic process of these species as far as referred in previous studies.

Line 83: LPF

This abbreviation should be explained at first.

Line 129-134: In this study, we aim to show…

There is logical gap between this paragraph and former description of Introduction.

Material and Methods

Line 164: Empty shells found in the…..

It is unclear how authors deal with cytoplasm bearing or empty shell sample in this study. Do these CT data of shells contain information about the presence/absence of cytoplasm in each specimen? Is it possible to compare the CT image of shells that are containing cytoplasm or not? It is also unclear how authors interpret the difference between presence and absence of cytoplasm, and how it is represented in the Result and Discussion.

Line 165-166: The complete description of sample…..

I suggest to add the description about surface sediment sample a little.

Results

Line 240-241: The water column can be divided into two……

What is reason for separating the water column at 75 m? And, is it really reasonable to divide water column into two layers? It is unclear for me how does this work in the following Results and Discussion.

Line 242: 4.0-3.0

Change to 3.0-4.0?

Line 249-250: *Turborotalita quinqueloba* reaches its peak shell density……

Is this statistically significant?

Line 251-252: The outer shell walls are thick and dense, while…..

This is just a comment. This is very interesting result. To me, it seems to be caused by inorganic dissolution of juvenile shell after shell calcification. But I have no idea why the shell can be dissolved in the water column such quickly. If this is due to the process of gamtogenesis, it must be very energy consuming ontogenetic process for foraminifera, because it is time consuming process to dissolve the calcified shell in the nature condition.

Line 270-277: Although we found…..(Paragraph)

It is unclear for me what is this paragraph intending. If the authors want to mention about the variation in the CT data, it might be worth to show with box plots or to consider whether there are outliers.

Line 311-327: 3.3.2 Planktonic Foraminifera from the surface sediments

As describe in major comments, it is better to explain why measurement of shell samples from the surface sediment is necessary.

Line 319-321: In *T. quinqueloba*, the loss of the base features of the prominent spines is evident as the…

I could not figure out this from Fig. 6.

Line 355: In addition, *L. helicina* shell density has an inverse relationship with ΩAr (Fig 8A–8B).

Is it possible that thinner shell wall is scanned as lower CT number due to the resolution of CT scanning? I don't mind this in the case of planktic foraminifera, because they are enough thick, but I'm wondering what about the case of pteropod with very thin shell wall.

Discussion

Line 411: Cytoplasm-bearing specimens are also present in the entire water column (S7 Table), …

Is it possible to compare the CT images of shell with/without cytoplasm individually?

Line 422: However, this is less likely in planktonic foraminiferal shells……

So, how can we interpret this? I thought that very low Ω water and long duration of time is required to dissolve matured calcite shell. But it seems definitely dissolve from inside of shell based on the CT images. I am interested in whether they have cytoplasm or after gametogenesis.

Line 455-456: The ability to build a thicker and denser crust may be explained by the difference in lifecycle length between *N. pachyderma* and *T. quinqueloba*.

This is very interesting suggestion to interpret the difference of shell dissolution tolerance between species. If this the case, are there significant difference between shell wall thickness or shell area density proxies?

Line 483: The increase in whorl thickness could be related to the ΩAr in the depth interval in which they live.

Why authors can say that shell thickness related to the ΩAr? Isn't it just representing that shell gradually thickens in the process of shell growth?

Line 508-509: The presence of these pteropod shells in the surface sediments confirms that…..

I could not understand why this can be an evidence for building thicker shell wall in low ΩAr

environment.

Line 511-522: With the ongoing OA in polar regions……especially lethal for juvenile *L. helicina* [33,35].

As shown in major comments, I'm afraid that the discussion of speculated reaction of *L. helicina* to ocean acidification described in this paragraph might be going beyond the scope of this study.

Figures

Figure 2:

Please unify the marker shown in chart and annotation. Because many dots are overlap on this figure, this figure is hard to see. I recommend to reorganize this figure. How about to use box plots? In addition, author should reconsider about color selection. Red and orange are hard to distinguish. It might be better to use other color or different marker each other.
