# Supplementary material for: Shell density of planktonic foraminifera and pteropod species Limacina helicina in the Barents Sea: Relation to ontogeny and water chemistry
Source: PLoS One. 2021 Apr 28;16(4):e0249178. doi: 10.1371/journal.pone.0249178 (PMC8081242; doi:10.1371/journal.pone.0249178)
Supplement: Attachment — Submitted filename: Response to Reviewers.docx [file pone.0249178.s007.docx]

Dear Editor,

We thank both reviewers for their constructive comments and feedback. Both B. Metcalfe and anonymous reviewer #2 suggest major revisions. We have corrected our manuscript following the reviewer’s suggestions and address each comment below. In the few cases, where no change in response to a particular comment is made, an explanation is given.

Yours sincerely,

Siri Ofstad, Katarzyna Zamelczyk, Katsunori Kimoto, Melissa Chierici, Agneta Fransson and Tine L. Rasmussen

**Reviewer #1**

Below we reply to the comments by Reviewer #1. The reviewer’s comment is in italics and our replies are in normal script. We thank the anonymous reviewer#1 for pointing out some weaknesses in our title and introduction, which have helped us greatly in sharpening the introduction and making it clearer. We also thank the reviewer for pointing out important and useful references and showing us other unclear parts of the manuscript – which we have now taken care of in this revised version of the manuscript.

Reviewer #1: Review of Relationship between shell density and vertical distribution of living planktonic foraminifera and pteropod species Limacina helicina in the Barents Sea

*This manuscript presents CT scans of planktonic foraminifera and pteropods from the Barents Sea. The title suggests that the study is focussed on morphological changes, namely shell density, through a vertical profile of the water column,*

**Reply**: The reviewer has an important point and we realize that we have not been clear enough here and that the title can be misleading. This is a study using a new method (CT-scanning) in a high-Arctic area and as such is a pioneer study with the purpose of exploring eventual environmental relationships and if there are any signs of ocean acidification. We have thus changed the title so it better reflects the study. “Shell density and vertical distribution of living planktonic foraminifera and pteropod species *Limacina helicina* in the Barents Sea: assessment of relationship to environmental conditions”.

*however, the study site is only 340m water depth with strong lateral currents from the North Atlantic. It is not intuitive to me why the authors would have selected this location if the title were to reflect the original objective of this study*.

**Reply**: The fact that the study area is a shelf sea with relatively shallow average depth can lead to misunderstanding. Usually, planktonic foraminifera live in the upper water column both in the open ocean and shelf seas, normally in the upper ~200 m, where each species has each own depth preference. Because they can change their depth habitat during their life cycle (i.e. deeper), most species usually avoid coastal and shallow areas <100 m water depth. Our previous foraminiferal fauna study from the same area (Ofstad et al., 2020) showed a clear and consistent vertical zonation of species and abundances that led to the present study of investigating shell density and the environment at the same location.

Indeed there are relatively strong currents in the Barents Sea, however, based on data from plankton tows, most planktonic foraminifera > 150 μm reach the mean ocean depth of 3800 m in 3 to 12 days depending upon shell weight and presence or absence of spines (Takahashi and Be, 1984). This observation implies that sinking foraminifera shells take one to eight days to reach 3800 m from 1400 m depth. In addition to that, Honjo and Manganini, (1993) reported that there was no offset of arrival time for foraminiferal shell fluxes at 1000, 2000 and 3400 m depth traps in the North Atlantic, indicating their rapid settling speed. If we assume a sinking speed of 1 km per day, it is unlikely that the PF have been transported far since it is a matter of hours before they reach the seafloor. With a current speed of 10 cm/s they would have moved just 1.5 km laterally before while sinking to 200 m water depth.

*There is no real story in this data. The data is poorly represented, in fact I’d go as far as to say misleadingly so, in order to derive any story at all.*

**Reply:** Admittedly, the introduction can also be misleading (as the reviewer points out below) and perhaps lead to expectation in the readers that the manuscript deals with the effects of ocean acidification on planktonic calcareous organisms, when instead we are investigating several topics where ocean acidification is included, but is not the only focus. We are testing if there are such effects (see reply below). We have also removed parts of the discussion text that were particularly speculative related to *L. helicina* and adaptations, to avoid misleading the reader.

*The story that is drawn, that pteropods adapt to ocean acidification by thickening their shell, is entirely unsubstantiated by the data presented here*.
**Reply**: We recognize that we took our interpretation too far. In the manuscript, we no longer elude to an adaptation against ocean acidification.

*I summarise some of my issues below*.

*Introduction. I found the introduction to be poorly written and often ambiguous, leaving the reader guessing at what is meant… e.g. what is meant by “positive relationship” between ocean acidification and food?*

**Reply**: We acknowledge that it is our mistake entirely and that our introduction could be misleading as it focuses on ocean acidification in high detail and could lead to the expectation that this paper is about ocean acidification only (see also reply above). Our earlier investigation of the distribution patterns of planktonic foraminifera faunas and pteropods and water masses, indicated there is a high seasonal variability and a clear zonation of planktonic foraminifera in the water column. This present investigation set out to explore shell density with a new technique versus depth to see if there is a pattern that can be related to environment (water chemistry) and if data on shell density could indicate if ocean acidification is taking place in this environmentally highly variable area and if there are species-specific trends in shell density.

We have therefore edited and shortened the introduction and made it more precise as to the nature of our investigation with references to previous results from the area. There has been recent and large environmental changes in the Barents Sea. Also, the Barents Sea is particularly vulnerable to acidification. All this is now included in the new ‘Introduction’.

We also clarify what we meant by a “positive relationship” between ocean acidification and food” – this is now in the discussion instead of in the introduction.

*Moreover, the authors display no critical assessment of previous research. I would have liked the authors to show that they had considered the limitations or caveats of past studies, especially an appreciation for the limitations of experimental/incubation studies verses field observations, rather than implying that all studies are equally robust. Ocean acidification studies are full of inconsistencies and opposed methodologies. We can all learn from what has and has not worked in the past, it is a real shame that the authors did not take this opportunity to say why their study would make an advancement*.

**Reply**: We have deleted this part of the manuscript concerning ocean acidification culturing experiments. The focus of our manuscript is not only ocean acidification, but a study of shell density to test if any patterns in change in shell density can tell us anything about how shell density varies in relation to the environment, hereunder acidity. Therefore, going into detail about culturing experiments is beyond the scope of our study.

*Method. For the foraminifera it is very clear that the orientation of the cross section, and how much of the inner shell walls are sampled, has an influence on the mean CT number. I suggest that this analysis could be more robust had the CT number only taken into consideration the shell wall of the penultimate chamber. i.e. inner shell walls may be reabsorbed during the forams life cycle and/or disproportionally sampled by the cross section. The final shell wall may still be growing. The penultimate shell wall, and only that part that has an outer surface, could arguably be a more consistent/robust measure than the mean of the entire cross section*.

**Reply**: The mean CT number is not derived from the cross section, therefore the orientation of the cross sections has no influence on what the mean CT number is. The mean CT number represents the entire shell – all of the inner and outer shell wall is sampled, not just the shell wall which can be seen in the cross sections.

We followed this suggestion from Reviewer #1 and isolated the outer shell wall of the penultimate chamber and measured the CT number of that section. We also isolated the final chamber. The results show that the CT number of the penultimate chamber shell wall does not differ considerably from the CT number of the entire shell. We measured the shells that are shown in figure 3 and 4, and summarize the results in the table and figure below:

**Table 1. Mean CT numbers of whole shell, versus penultimate chamber and final chamber**

| Sample ID | Mean CT# of entire shell | CT# of penultimate chamber | CT# of final chamber |
| --- | --- | --- | --- |
| *1102 50 q5* | 726,1 | 698,0 | 740,9 |
| *1102 100 q8* | 797,1 | 826,5 | 798,2 |
| *1102 150 q14* | 848,3 | 833,6 | 846,1 |
| *1108 200 q94* | 813,9 | 836,6 | 802,6 |
| *1108 50 p62* | 631,6 | 627,8 | 634,2 |
| *1108 150 p73* | 796,4 | 814,0 | 783,8 |
| *1102 300 p48* | 842,9 | 832,1 | 839,4 |

|  | whole | penultimate | ultimate |
| --- | --- | --- | --- |
| 1102 50 q5 | 726.1 | 698 | 740.9 |
| 1102 100 q8 | 797.1 | 826.5 | 798.2 |
| 1102 150 q14 | 848.3 | 833.6 | 846.1 |
| 1108 200 q94 | 813.9 | 836.6 | 802.6 |
| 1108 50 p62 | 631.6 | 627.8 | 634.2 |
| 1108 150 p73 | 796.4 | 814 | 783.8 |
| 1102 300 p48 | 842.9 | 832.1 | 839.4 |

**Fig 1. Relationship between mean CT numbers of whole shell, versus mean CT number of penultimate chamber and final chamber of seven planktonic foraminifera.**

Based on this, we do not think that using the penultimate chamber is more robust than using the whole shell.

*Results. I took the time to look at the excel file of data supplied as suppplement. I was unable to reconcile the values in the “estimate” columns in tables S4 and S5 with the raw data. What is “estimate”? Furthermore, the units in these tables do not make any sense… I am pretty sure that the shell thickness is not >40000 mm!*

**Reply**: Thank you for making us aware of these mistakes in the supplementary file. The unit of the shell thickness has been corrected, and the “estimate” was the estimate of the slope, the name of the column has been changed to slope to avoid confusion.

*I am not an expert in stats, but I know enough to understand that the stats do not really support the statement on line 280 or any of the following conclusions. The r2 values are nothing to write home about. I suspect that had a deeper water site been chosen, there may be more of a story in the vertical profile variability*.

**Reply**: The r2 value represents the scatter around the regression line. Therefore, the low r2 can be attributed to the range in shell density we see in all five sampling intervals (see Fig 2). This does not change the fact that there is a correlation between the mentioned physical parameters and water depth. In our previous publication (Ofstad et al., 2020), we showed that the planktonic foraminifera at our 340 m deep study site have a depth zonation, making it sufficiently deep to do a study where water depth is an independent variable.

Line 285. *It is revealed that up to 85 % of specimens recovered from >150m water depth were “not living”. There is no mention of these specimens being dealt with differently/isolated from statistical analysis. Rather, in line 291 and again in the discussion, statements like “gradual shell thickening with depth” with no further recognition of the fact that many/most specimens are dead and therefore their position in the water column is more likely to reflect settling out/reworking by lateral/upwelling currents and/or “reworking” by predators. Once you’ve revealed that the specimens are not living it becomes a nonsense to draw conclusions are the life stages/depth migration of these specimens*

**Reply**: This is correct, but only for *T. quinqueloba*. 75% and 78.6% of the *N. pachyderma* shells contained cytoplasm in the two deepest sampling intervals. Including all shells, with and without cytoplasm in our analysis still gives valuable information. In the manuscript we now acknowledge that the empty tests could mean that they have been upwelled – rather than very recently died and settling to the seafloor.

Line 292+ *Foram shell thickness of 2-4 microns? I suspect that is an order of magnitude out. Had the authors looked at other seminal works on N. pachyerma sinistral they would have known that there measurement of shell thickness was way out. Kohfeld and Kozdon references are amongst critical references omitted from this manuscript*.

**Reply**: We thank the reviewer for pointing this out and making us aware of the key references we have missed. These references are now included in the manuscript.

The mean shell thickness of the shells is indeed lower than expected; this is due to the method used to derive the average shell thickness (calcite volume/surface area). The calculated thickness parameter does not always represent the actual shell thickness. This is because the thickness parameter also takes the porosity of shell into account. For example, a porous shell contains many voids (can be seen in Fig. 7E+F), resulting in an increase in surface area. In this case, the actual shell thickness does not change but the calculated thickness will decrease. This caveat has now been clarified in the manuscript. With that being said, the living forams are not dissolved (Fig 3+4), and there is a clear relationship between the CT number and the calculated thickness (Fig 5). Therefore, we think it is reasonable to assume that the calculated thickness has a linear relationship with actual thickness.

*Line 295. What is μm-m?*
**Reply**: This has been removed.

*Line 300. This is the ONLY parameter compared with shell thickness!*

**Reply**: We have also added a comparison between CT number and shell volume in the manuscript and supplementary information. Additional parameters could be added, however the entire data set with all physical parameters measured by the XMCT is included in the supplement should anyone want to test other parameters. It is also to be expected that shell thickness is the parameter that correlates the strongest with shell density (CT number), therefore we do not think it is necessary to include all of the comparisons in the manuscript.

*Line 324. What is this 2-layer dissolution pattern? I don’t see it in the figure.*

**Reply**: The two-layer dissolution pattern is referring to the divide between the less dense (uniformly dark blue) inner calcite and the more dense (light blue/green/orange) outer crust. This explanation has been added to the text to clarify what we are referring to. See a more zoomed in image of the *N. pachyderma* in Fig. 7F.

**Figure 2. Two-layered dissolution pattern found in *N. pachyderma* from surface sediments, 10 micron scale.**

*Line 355. Because Ω varies with depth!*
**Reply**: Yes, this is correct. To properly define the relationship between the vertical distribution of *L. helicina* in the water column, their shell density and Ω_Ar_ a more extensive investigation needs to be done. But by stating the apparent inverse relation between Ω_Ar_ and shell density we carefully speculate (not concluding anything), that these two could be linked. The need for longer studies is highlighted in section *4.2.2 Dissolution of L. helicina, ontogeny, and future outlook*.

*Line 357. The authors conclude that shell thickness of L. helicina increases in sequential whorls. This is absolutely not supported by the CT images. i.e. the thickness of the shell whirls shown in figure 8f do not support the plot shown in 8d. The only part of the shell that shows anything like the thickening reported in the plots is the structural part of the shell at the central spiral which must increase with each whorl for structural reasons. There is absolutely no evidence to support thickening of the shell as an adaptation to ocean acidification. This is a poor attempt to drag a story out of this data. The discussion from line 483 onwards, based on these ill found conclusions, is completely unsubstantiated.*

**Reply**:

Figure 8D is now shown as a boxplot as the previous bar chart was a poor choice for showing the increase in whorl thickness, and that the differences in the means is significant. Figure 8F shows where we did the four measurements of the shell apex. This part of the shell appears to thicken uniformly at all four points when the number of whorls increases, the above shell has 2 whorls while the below shell has 2.7 whorls (Figure 8F). A Mann Whitney U test was used to test the significance this (shell apex measurements from *L. helicina* with 1.5-2.25 whorls vs shells with 2.5-3.5 whorls), which is shown in the boxplot 8E. The individual whorl thickness measurements were done, as you pointed out, on the central spiral. As we have mentioned in the previous comments we have toned down our interpretations of this data. Our data does however show a statistically significant increase in *L. helicina* shell density with depth between the 0-50 m depth interval and the underlying sampling intervals (Table S10).

**Reviewer #2: B. Metcalfe**

Below we reply to the comments by reviewer #2, B. Metcalfe. The reviewer’s comment is in italics and our replies are in normal script. We thank B. Metcalfe for encouraging more statistical analysis, thorough examination of the supplementary materials and comments pertaining to the figures. We also thank the reviewer for pointing out important and useful references which have strengthened the manuscript.

Major comments:

*There is some missing information from the methodology which should be elaborated upon in this paper. For instance the authors have weight (supplementary table 4 and 5) but no mention of how it was measured/calculated (what device, in groups, individually?). Were the foraminifera picked from >63 um? From a recent publication the authors picked from 150-200 um, are these the same specimens or were the specimens for isotopes picked first? Etc. etc. Please expand the methods section.*

**Reply**: The methods section has been expanded and now include all that is suggested by Reviewer #2. We have added the sampling protocol for the foraminifera and pteropods. In addition, we provide more information on the parameters thickness, volume, surface area measured by the XMCT.

*From the mean CT number you can have variation either from the total number of voxels with a specific ct number or with voxels from the whole test. I.e., the ct number can apparently reflect size change or thickness/volume change. The authors do point out that size variation can disrupt their data (“outliers may occur if specimens have an unusual morphology, as for example in the T. quinqueloba plot, there is an individual with an abnormally large final chamber, which also has a low density (Fig 5B)”). Have you considered further normalising each ct number by the size of the shell to remove the potential change in size with depth complicating your results? I do wonder if this already done in the ‘thickness’ measurement which is volume/surface area, which to me sounds more like the volume of calcite normalised to size rather than a measure of the thickness of the shell.*

**Reply**: We believe that one of the reasons why the XMCT is such a robust method is because it does not require any size normalization. The CT number is already a mean, and would therefore not cause any kind of inflation of CT number as the shell size increases. We think it would be wrong to remove the influence of size on the shells because larger shells are usually more dense (p = 0.002). We believe it is valuable to show the large variation in shell density and size with water depth (shell diameter with depth can be seen in Ofstad et al., 2020). The change in shell size with depth is not statistically significant (Tabel S4-6), which is also a reason not to do a size normalization. However, because of another comment of reviewer B (see below) pertaining to the distinct 2-layer Ω profile, we did do a test on size normalized shells (CT number/volume) living in the upper layer (0-50 m) and those found in the lower layer (50-300 m). The results from the test are included in the manuscript.

*Line 42-44 ‘We show that the density of L. helicina shell has an inverse relationship with ΩAr and hypothesize that the gradual thickening of the shell wall could be an adaptation against low ΩA’. I don’t actually see where this is directly compared (apart from plotting in Figure 8 a panel with the ΩA values next to a panel with CT number), there is a comparison with depth (supplement table 6) but no direct comparison (r2, p value) between ΩA and CT number****. Please add this into the results section*** *(line 355). Furthermore, in the supplement I don’t see any tests against environmental variables, whilst most environmental parameters covary and most changes can be seen with depth, the authors have chosen only to test depth. Whilst culture studies have shown a relationship with carbonate parameters, from your data you could also argue that shell density has an inverse relationship with temperature.*

**Reply**: Thank you for this comment. Tests have now been done for carbonate ion concentration and temperature (see Table S4-6), in addition to ΩAr for *L. helicina* (Table S6). The measurement was taken from the midpoint of the sampling interval, if we did not sample at the midpoint we calculated the average between the maximum and minimum depth of the sampling interval (e.g. we do not have a ΩAr measurement from 75m, so we averaged the measurement from 50m and 100m). The p-value and r^2^ between the *L. helicina* CT number and ΩAr are now included in the results section for *L. helicina*.

*Out of curiosity what is the rationale for plotting the maximum depth of the net interval as opposed to the mid-point? As depth is an explanatory variable and tested rather than environmental parameters why choose the deepest depth? How does shifting the depth to the minimum or midpoint impact your r2.*

**Reply**: We chose to plot the maximum depth because it is the depth at which the net opened, and the deepest point that a shell could have been sampled from. Plotting with the midpoints or the minimum point are not more correct in our opinions. But to ensure that there is no confusion we have added the sampling intervals to the y-axis of all vertical plots.

To test if there is a significant difference between using the max, midpoint or minimum depth in the sampling interval, we did a linear regression on the different water depths and CT number of *N. pachyderma*.

|  | Intercept | Slope | Std Error | t-value | p-value | r² |
| --- | --- | --- | --- | --- | --- | --- |
| **Water Depth (max)** |  |  |  |  |  |  |
| CT Number | -277.6388 | 0.5958 | 0.0960 | 6.207 | 8.48e-09 *** | 0.25 |
|  |  |  |  |  |  |  |
| **Water Depth (midpoint*)** |  |  |  |  |  |  |
| CT Number | -261.69599 | 0.53374 | 0.08595 | 6.210 | 8.35e-09 *** | 0.25 |
|  |  |  |  |  |  |  |
| **Water Depth (min**)** |  |  |  |  |  |  |
| CT Number | -245.75323 | 0.47166 | 0.07677 | 6.144 | 1.14e-08 *** | 0.24 |

**Table 2. Statistical comparison between different water depths in sampling interval against mean CT numbers of *N. pachyderma* using a linear regression model. *Midpoint: 25m, 75m, 125m, 175m and 250m. **Minimum: 0m, 50m, 100m, 150m, 200m**

The r^2^ is 0.01 lower when using the minimum depth rather than the midpoint or the maximum which we use in the manuscript. We will therefore keep using the maximum depth in all statistical analysis.

*In section 3.1 you identify two distinct layers, could you not test statistically the difference between shells in the upper layer and the lower layer. Normalising for size change to see if they are differences which are statistically significant.*

**Reply**: This distinct layer of elevated Ω saturation is at 0-50 m. The difference in shell density between the shallow layer (0-50 m) and everything below (50-300m; which is quite homogeneous in terms of Ω) was tested for significance with a Mann-Whitney-U test. The CT numbers were also normalized for size (CT number divided by calcite volume), as you have suggested. We summarise the results below:

- The size normalized CT number of *N. pachyderma* from the upper (n = 19) and lower (n = 101) water column showed a significant difference, p-value = 0.004033.
- The size normalized CT number of *T. quinqueloba* from the upper (n = 24) and lower (n = 91) water column showed a significant difference, p-value = 4.414e-05.
- The size normalized CT number of *L. helicina* from the upper (n = 6) and lower (n = 19) water column did not show a significant difference, *p-value = 0.2465.*

This result has been added to the manuscript. Although they are both statistically significant, the very small p-value from *T. quinqueloba* compared to *N. pachyderma* highlights that *T. quinqueloba* has a stronger shell density zonation through the water column.

*Whilst it’s not a major comment, the use of LPF instead of living planktonic foraminifera seems to be an unnecessary acronym e.g., line 473 ‘This scenario may be found when LPF calcify in low...’ do you need ‘living’ in this sentence? It would be precipitation rather than calcification if they were dead. Or line 524-526 ‘living planktonic foraminiferal (LPF) species Neogloboquadrina pachyderma and Turborotalita quinqueloba and the living pteropod species’ extant or modern would be the preferred terms.*

**Reply**: The abbreviation LPF has been removed from the manuscript and replaced by PF (planktonic foraminifera) to avoid confusion.

*Finally, I have tried to note down lines in which the references need to be adjusted to fit PLOS’s reference style, these are: 65-66; 90-91; 151; 154; 166, 212, 578.*

**Reply**: Thank you for making us aware of this. They have all been corrected to the proper reference style.

Minor comments:

*Line 60: ‘Also, the cold surface water has greater CO2 solubility’. Personally I would put a rephrased version of this (e.g., ‘The solubility of CO2 in seawater has a relationship with temperature’) at line 57 just after ‘ocean acidification (OA).’*

**Reply**: The introduction has been rewritten to not be as centered around ocean acidification as it was previously. This is because we do not actually see any effects of ocean acidification on the forams or pteropods, and the water chemistry is not particularly corrosive. The old introduction could have been interpreted as misleading for some readers. Therefore we saw it more fitting to have the introduction more focused on the Barents Sea, the importance of foraminifera and pteropods, and a bit of background information on ocean acidification and what information we get from shell density studies.

*Line 61 remove ‘that’, replace ‘is’ with being : “results in the Arctic Ocean being particularly”*

**Reply**: The introduction has been rewritten and sentence deleted in the process

*Line 62-66: clarify for the reader whether this is only surface waters or includes intermediate / deep (give the potential depth habitat of foraminifera, pteropods etc)*

**Reply**: same reply as above

*Line 68 ‘and cause’ replace with ‘with an associated’*

**Reply**: The introduction has been rewritten same reply as above

*Line 69: ‘the observed ocean’ – are you still talking about the Barents Sea here or the Arctic Ocean?*

**Reply**: same reply as above

*Lines 66-73: You can condense this section, e.g., ‘The Barents Sea is one of the largest CO2 sink areas in the Arctic region, which is 67 mainly caused by the year-round CO2 undersaturation and high biological production [13,14] despite the formation of sea-ice in winter. The Barents Sea CO2 sink is predicted to double by 2065 with an associated a pH decrease of up to 0.25 pH units [15]. A significant proportion of the observed ocean CO2 increase between 1967 and 2001 of 42 μatm has been from the inflow of Atlantic water, which is rich in anthropogenic CO2 [16]. The meltwater from sea ice or glaciers lowers the ΩAr and ΩCa [17–19], and is predicted to increase as a result of warming [20].’*

*Line 83 define LPF (‘There is no known ΩCa thresholds for LPF’) rather than in lines 163 or 393 and is this sentence a repeat of the line 85 (‘There is no known ΩCa thresholds for planktonic foraminifera’)? Regardless the word ‘is’ should be ‘are’ as you are not talking about a singular thing.*

**Reply**: Done, section has been condensed following the outline above.

*Line 91-93: I would modify the rationale a bit (i.e. directly answer why it’s necessary to have in situ studies if culture studies have shown X – this also links back to your biomonitoring in your abstract) : ‘Having demonstrated the impact of lower CaCO3 saturation states in culture, it is important to obtain in situ data for comparison and to put culture experiments into context through attempts to link species biological response to changes in ocean chemistry.*

**Reply**: The introduction has been rewritten and we took out references to ocean acidification studies and methods

*Line 98 replace ‘but this’ with ‘a similar’. Or remove this section and add it to the end of line 101.*

**Reply**: The introduction has been entirely rewritten, therefore not applicable anymore

*Line 105: ‘There is a lack of established methodology to study biological effects of OA.’ The subsequent discussion is with respect to ‘calcification’ perhaps alter this sentence to reflect this?*

**Reply**: same reply as above

*Line 110 ‘have become better refined and accurate over time’*

**Reply**: same reply as above

*Line 163: I don’t see the necessity in using LPF if it reflects both empty and cytoplasm bearing shells (i.e., it would be better to use when only referring to cytoplasm bearing foraminifera)*

**Reply**: Agreed, we have omitted the acronym LPF

*Line 166: I would include all pertinent procedures and chemicals i.e., from the reference given, give a brief version of the plankton tow and sediment procedures. I.e., repeat and rephase both “*

*Immediately after recovering the sediment core, the top layer (1 cm) was scraped off by a*

*spoon, and preserved in approximately 50 ml of ethanol (96%) with rose bengal (2 g L−1 of*

*ethanol), and stored at 2 °C.”*

*... and “the samples were sieved with sea water through a 63‐μm sieve and transferred into*

*plastic bottles (250 ml) where they were immediately fixed and buffered with approximately*

*230 ml ethanol (98%), a quarter of a teaspoon hexamethylenetetramine (≥99.0%), and stored*

*at 2 °C. Once returned to the home laboratory, the samples were briefly washed with tap water*

*on a 63‐μm sieve in order to remove organic particles from the surface of the foraminiferal*

*tests and to break up aggregations of material.”*

**Reply**: Done, summary of sampling methods has been included.

*Line 166: I would include some information of how you selected the specimens. Were all foraminifera picked from >63um? Were the shells picked ‘randomly’ or a selection of specimens ranging in size? In Ofstad et al., (2020) the foraminifera were picked from 150-200 um is this the case here?*

**Reply**: This is a good point. We have included more information of which specimens were scanned by the XMCT. It is also acknowledged that this could cause a skew in the vertical density profile with depth. The 150-200 micron size range in Ofstad et al., (2020) is only referring to the foraminifera that were measured for isotopes.

*Line 184-186: Dividing the volume by the surface area to calculate the thickness, doesn’t this massively over simplify the thickness? To me it sounds more like the volume normalised to shell size than ‘thickness’ of the shell. How is the surface area chosen of a 3D object, is it the maximum surface area?*

**Reply**: Yes, this is correct, it is an over simplification and because of that we have to modify our interpretation of the calculated mean thickness. The calculated thickness parameter does not always represent the actual shell thickness. It also takes the porosity of shell into account. For example, a porous shell contains a lot of voids (can be seen in Fig. 7E+F), resulting in an increase in surface area. In this case, the actual shell thickness does not change but the calculated thickness will decreases. This will be clarified in the manuscript. With that being said, the living forams are not dissolved (Fig 3+4), and there is a clear relationship between the CT number and the calculated thickness (Fig 5). Therefore we think it is reasonable to assume that the calculated thickness has a linear relationship with actual thickness. The outliers (cross symbols) are from the sediments and have all experienced some degree of postdepositional dissolution which has altered their surface area. The surface area of the shells includes all surfaces which are exposed to the air, this includes not only the outer areas, but also the surfaces of the internal chambers.

*Line 191: “Live (cytoplasm bearing) planktonic foraminifera specimens were counted for each depth” seems out of place here in the XMCT section, perhaps move to prior section/ line 166*

**Reply**: Done, moved to marine calcifiers section

*Line 211: ‘nearest 10th’, does this mean that your whorl measurements are rounded to ~18 degrees (half of 36)? Include Janssen’s (2007) point that the determination of “estimating the ultimate whorl” has “an accuracy of a quarter whorl”.*

**Reply**: Whorl count data is corrected to be to the nearest quarter, following the Janssen (2007) methodology.

*Line 215: have you determined what is the error associated with the adobe ruler tool? E.g., repeat measurements of the same shell wall? From experience with Photoshop ruler, ImageJ and all manner of other measurement software I have determined thickness, shell size, etc from a couple of measurements just to average out potential undulations in wall, etc.*

**Reply**: Thank you for pointing this out. Repeated measurements were done and has been added to the methods section.

*Line 256: missing ‘–‘ between 200 and 300 m*

**Reply**: Corrected

*Line 247-256 / Line 270-277: The authors have brought up that their increase should be viewed in light of the range in depth (“Although we found a general increase in CT number and shell thickness with depth, we note a large range in CT numbers (Fig 2 and S2 Table) and mean shell thickness (S2 Table) at each sampling depth interval.”), I agree with them, have they considered testing whether the difference between depths is statistically significant? Rather than testing the significance of the linear fit, testing whether the samples within each net are statistically different from one another. Plotting the min, max, mean with std dev as error bars from table s2 it does seem apparent that there is a greater shift in the minimum than the maximum for the ct number.*

*OR plot the raw data as four panels in boxplots for figure 2 (see figure comments below)*

**Reply**: Thank you for the suggestion. A Mann-Whitney-U test was performed for each depth interval for the CT numbers of *N. pachyderma*, *T. quinqueloba* and *L. helicina* (see results in S8-10 Table). In summary, and not surprisingly we found that the significant differences were mainly between the shallowest (0-50m) and deepest (200-300m) sampling interval. *T. quinqueloba* showed the most significant variation between nets, and *L. helicina* the least.

Figure 2 has been redone so the data is displayed as boxplots in three different panels. A fourth panel with all of the data together was not made because it was far too messy.

*Line 280: “Both N. pachyderma and T. quinqueloba show a statistically significant positive correlation between individual shell weight, CT number, mean shell thickness and area density with water depth (S4–S5 Table)”. Where are the intercepts in the supplementary table? The ‘estimate’ is the slope, no?*

**Reply**: This is correct, the estimate is the slope. We have changed the name of the column from estimate to slope to reflect this. The intercept for each variable has been added to S4-6 Table.

*Line 346: In your supplementary table (limacine helicina tab) I am confused about how the numbers of whorls corresponds with measurements labelled whorl 1, whorl 2, whorl 3, whorl 4. For instance how can a shell with 0.6 whorls have measurements in whorl 1 and whorl 2 column? Especially if the shell in fig s3 has 3.5 whorls and has four measurements. The same for 1.3 whorls having measurements in whorl 1, 2, and 3 columns (there is also a discrepancy some 1.2 whorl shells have 3 measurements some have 2). I.e., how does a shell have measurements for whorls it does not have (or have I read this wrong?).*

**Reply**: No, you have not read this wrong. Whorl 1 should have been referred to Whorl 0, which is the protoconch, and Whorl 2 should be Whorl 1 etc. The measurement of shell wall thickness is done in between two whorls, i.e. the measurement for Whorl 4 is done approximately at 3.25 (Figure S3). The naming of the whorls has been corrected in the supplementary tables, figures and text.

*Line 356: A further question, looking at the supplementary table, is how comparable is a whorl measurement made on X.3 of a whorl compared with say X.8. In figure S3 your stars seem to avoid the shell opening (understandably given the difficulty in obtaining a reliable measurement there), but I assume with a smaller amount of whorl grown the measurement of shell thickness has to be made closer to the opening of the shell. Have you tried to estimate the impact of this on your measurements? Is some of the variation between the same whorl number but for different shells partly to do with how close the measurement is made to the opening of the shell?*

**Reply**: We made the measurement at the same location (see Fig 8), where the shell wall “dips” and forms a U-shape. The measurement was done in the middle of the U. Naturally there are small variations between each shell we measure, but we repeated the measurements three times and made sure not to deviate from this “dip”.

*Line 349: In your supplementary table there doesn’t appear to be a shell with 3.1 whorls, at 200-300m (the only corresponding values are those made on shell 1108 200 pt14 with 2.8 whorls), and the measurements of the zoomed in section for the 1.8 whorl shell comes from a shell with 2 whorls in the supplement.*

**Reply**: The larger shell in the cross-section is indeed pt14 200, which is the second deepest whole shell you see in Fig 8B. The black lines in Fig. 8B and C have been changed to reflect this, in addition to the caption. For the smaller shell, the discrepancy between 1.8 and 2 whorls is due to a re-count of whorl numbers, and forgetting to update the caption afterwards.

*Line 351: indicate in the caption what type of ‘error bar’ the lines on the bar chart refer to (standard deviation, standard error, CI?). And what are you plotting here, the mean?*

**Reply**: The data in the bar chart has been replotted as a boxplot (Fig. 8D), therefore this comment is no longer valid.

*Line 361: ‘while shells with less or equal’ , your supplementary table uses ‘equal less than 2’ (with data = 0.6-2.0) and ‘greater than’ (with data = 2.0-3.6). Check if you mean 2 or 2.3 and check whether shell ‘1108 100 pt3’ is missing a decimal place (otherwise it should be in the equal or less). I would reorder the supplement so that it corresponds with the value quoted in the text . Furthermore, as you have data for the top measurements only from shells with 1.5 -2.3 and 2.3-3.6 whorls, would it not be better to word it as such?*

**Reply**: The figure is correct, we separated the data into >2.3 (now 2.25 because the whorl counts are done to the nearest quarter), and equal or <2.3. The supplementary table now reflects this, and the figure has been corrected, i.e. 2.3 is now 2.25. Thank you for the suggestion to reword the top measurement categories to “shells with 1.5 -2.3 and 2.3-3.6 whorls”, this has been implemented.

*Line 359-362: Could you explain the rationale for using the sum of the four values as opposed to say the arithmetic mean? Likewise what is the rationale for splitting the measurements into < 2.3 whorls and >2.3 whorls? Was this related to the number of samples or does it have a biological/morphological rationale? Whilst the authors should check (see previous comment) if they mean 2? What would happen if you split the dataset in a different way.*

**Reply**: There was no rationale behind that decision, but the outcome would be the same if we used the mean instead of the sum. This was tested and the difference between the two groups was still statistically significant (p-value = 0.0021) when using the mean instead of the sum. We made the split at 2.3 whorls so there would be an almost equal number of shells that had >2.3 whorls (n = 9) and <2.3 whorls (n = 7), this would give the fairest comparison. When the dataset is split at 2.5 whorls, the difference is still significant (p-value = 0.00025). Also when the dataset is split at 2 whorls (p-value = 0.019).

*Line 360-362: “The sum of four measurements done on the central-top part of the shell show that...” (replace done with made)– Are some of these four measurements from the same whorl? Its hard to tell with a 3D image, perhaps indicate on figure S3 so that a reader can see how the stared thickness measurements correspond with these top measurements. In the limacina helicina supporting information why do some have Top 7; Top 6; Top 5? What do these additional measurements correspond with?*

**Reply**: Yes, two wall thickness measurements of the top of the shell are done of the same whorl, with the exception of the protoconch (Whorl 0). The precise location that these measurements were made is now indicated, as suggested, on Figure S3.

*Line 400: The North Pacific data of G. bulloides uses area density and outer wall thickness, so the percentage change might not be equivalent. Have you considered computing the same methodology as ref 31 (Iwasaki et al., 2019), (1) to see if the methodologies are comparable and (2) to directly compare the values here.*

**Reply**: Done – we calculated the area density (weight/surface area) of the shells that were weighed (111 in total). The percentage change, on average, for each depth interval was calculated and added to the results text.

*Line 405-408: Could the changes also reflect the fact that the species is spinose (http://www.mikrotax.org/pforams/index.php?id=104250). So differences between non-spinose (N. pachyderma) could reflect different calcification mechanisms*

**Reply**: Thank you for the suggestion. We agree, and have added that to the beginning of the section (4.1.1 Comparison N. pachyderma and T. quinqueloba in the water column and their preservation patterns)

*Line 409-412: ‘If we use thick density shells as a proxy for reproduction’ Why not use the appearance of non-cytoplasm bearing shells as a proxy?*

**Reply**: There are biological factors that may cause premature mortality, and hence the loss of the cytoplasm. Also, we must consider human errors, especially when it comes to *T. quinqueloba*. In the absence of symbionts, *T. quinqueloba* may have a transparent and thin cytoplasm, making it extremely difficult to determine whether a cytoplasm is present or not (Schiebel and Hemleben, 2017). Therefore, we do not see it as a suitable proxy for reproduction. It is also possible that the cytoplasm was lost after sample collection. But we acknowledge that a particularly thick shell does guarantee that it has undergone gametogenesis. It is unknown whether or not a fail-safe physical characteristic for gametogenesis exists.

*Line 417: some early culture papers suggested that wall dissolution could be a common occurrence*

**Reply**: Thank you for making us aware of this. Two references have been added to the text which are both culture studies on modern plantic foraminifera that show that dissolution starts on the inner part of the shell, and suggest that this could be a common occurrence. The references are Brown and Elderfield, 1996 and Hecht et al., 1975.

*Line 420-424: What about the sinking of dead shells, if as Takahashi and Bé suggested in their sinking speed studies sinking speed is related to shell size/weight. Then the decrease in density at the deepest nets could also reflect a relic population (or some specimens of such a population) that haven’t sunk far enough yet.*

**Reply**: Thank you for catching this. The time it takes for a shell to sink from the surface to the bottom (which is a little of 300 m in our study area) would be about one day. You are right, the less dense shells will sink slower, but we do not believe that the effect will be significant enough to leave behind a “relic population”. If we look at Figure 3, the density shown by the CT number of the internally dissolved *T. quinqueloba* shells and “pristine” ones in the shallower depth interval is only, on average, 20. Some of the internally dissolved shells could also have been larger in size, making this difference in CT number negligible on the sinking speed. However, this is a valuable comment and included in the manuscript as a possibility.

*Line 447: replace fragments with fragmentation*

**Reply**: Done

*Line 454 - 461: “The steeper N. pachyderma curve suggests that they continue to calcify for longer than T. quinqueloba” whilst its true they seem to have calcified more it’s not possible to extract a rate or length of time. I would avoid extrapolating too much from your dataset, your data comes from several hours so its next to impossible to really test the arguments made here. The same data could be used to argue a longer life cycle just as much as it could be used to suggest that the species calcifies more often; produce more calcite per calcification event (maybe spinose species invest more time building spines), etc.*

**Reply**: The suggestion of a timescale has been removed from the sentence.

*Line 470: ‘shells which have undergone either post-depositional dissolution or calcified in low ΩCa waters’ – include reference 46 (Johnstone et al., 2010) here as this is an idea very similar to what they proposed*

**Reply**: The above reference has been added.

*Line 473-474: “This scenario may be found when LPF calcify in low ΩCa waters, and shift their ecological strategy to favor shell size over shell density [31].” – Although T. quinqueloba is known to have a quirky shell outline with considerable variability of form related to multiple environmental parameters (if you can get access to it, see ‘Phenotypic variation of Turborotalita quinqueloba (Natland) tests in living populations and in the Pleistocene of an eastern Mediterranean piston core’, D Kroon, PF Wouters, L Moodley, G Ganssen...et al).*

**Reply**: This is true, nice suggestion and interesting paper. Added to the text.

*Line 493: ‘and as long as the ΩAr is sufficiently high’ – you could replace sufficiently high (is 1.2 high?) with ‘and as long as ΩAr is sufficient (>1.2)’*

**Reply**: Done

*Line 501-510 and 511-522: Some of the discussion points raised in these sections go beyond the specific study and would necessitate a longer targeted study (e.g., either long term plankton tows or sediment trap studies) to answer them all (e.g., “The sampled L. helcina population at the crater area most likely were spawned”; “ they will prioritize increasing their thickness and density in preparation for fall/winter when they most likely will experience a minimum in ΩAr”; “We speculate, that the decrease in ΩAr may lead to veligers”; “the veligers may start producing thicker and denser shells relative to what we see today”). Therefore, this would be a good point to bring in one of the conclusions raised in the abstract, lines 45-47 (‘This study clearly demonstrates the importance of biomonitoring to understand the variability in shell density of calcifying zooplankton’) that has so far not been directly raised. Perhaps rephrase the start along the lines of ‘A longer study using the techniques described here could answer....’*

**Reply**: We agree that these two paragraphs are highly speculative, and should instead be examples of potential (longterm) studies using the methodology. This section has been cut down and rewritten.

*Line 524-525: living is redundant here, extant or modern etc. Do you mean specimens rather than species here?*

**Reply**: Done, ‘living’ has been replaced with ‘extant’. I mean species because I’m referring to the species that were used in this study, i.e. *N. pachyderma* and *T. quinqueloba*. Indeed, specimen from the species were scanned, but I think the sentence is comprehensible.

*Line 565 ‘sampling’ is missing a p*

**Reply**: Corrected

*Line 570 remove ‘on’*

**Reply**: Removed

**Figure comments**

*Figure 2: The N. pachyderma data point at 200 m that is below or around 600 in CT number, this datapoint is lower than the value in table S2 for this species. Please check this datapoint. You have a minimum value for 484 for T. quinqueloba but your x-axis starts at 500. I would double check if merging the three plots hasn’t led some datapoints to shift.*

**Reply**: As mentioned in the “minor comments” section, Figure 2 has been redone and is now three separate boxplots. The x-axis for *T. quinqueloba* has been extended to start at 480.

*Figure 2: A lot of the visual impact of your data is lost by plotting all of the data together, why not plot as four panels (i.e., single panel for each species and a final fourth panel for the combined plots). By splitting the species specific data you could also indicate which specimen came from which plankton tow. A boxplot for this data (you can plot the raw data as a transparent overlay) would also give the reader an indication of the structure of the data (i.e., quantiles, median, etc). At the very least it is important to plot the min, max, mean, std dev so that readers can see the data in a clearer way.*

**Reply**: You are quite right, all of this information including the raw data is now in Figure 2. See above comment.

*Figure 3/Figure 4 Panel C: Plot the standard deviation of each mean. It is a nice visual plot, but I wonder why is the calcite saturation and depth ‘undulating’ , i.e., follow the lines. If there is suppose to be distance I would plot it. Furthermore, I understand 3 tows were combined but your plotting the mean of each depth vs. the variability in saturation along a transect. That does limit the comparison (we don’t know which shells came from which tow), especially for depth interval 50-100m where there seems to be the ‘largest’ variation.*

**Reply**: The standard deviation of the mean CT numbers has been added to Figure 3C, Figure 4C and Figure 8C. The calcite saturation plot that is used in the C-panel is taken from a larger cross-section plot. Because the distance between the three tows is so small and the water chemistry is more or less identical (see Fig S1 for salinity and temperature), we do not see it necessary to split the three tows when presenting data. Yes, there are some contours that vary slightly, but it is insignificant, and we believe it would only be confusing to add distance on the x-axis. The calcite saturation for all three stations is presented in the supplementary information table

*Figure 8D – I think you loose information by plotting it as a bar chart rather than as a box plot like E. The bars are simple so you can scale it down to fit with your figure so I understand why, but my problem is you can only compare the mean (? point out what the bars correspond to and what the error bars represent in the figure caption) all the information is lost about the lower end of each distribution (e.g., are there shells whose 4th whorl have thicknesses similar to their 1st whorl?)*

**Reply**: We have changed the plot in Figure 8D to be a boxplot like Figure 8E, thank you for the suggestion.

*Fig. S1: Add in the temperature and salinity datapoint for 300 m. You’ve plotted the mid points of the first and fifth net interval, I would do the same for the other intervals. This goes for Table S1 and S2, you have only given a temperature without indicating whether it’s the minimum or maximum depth. I would include the environmental parameters at min, mid and max depth intervals so that future researchers could incorporate your work into a meta-analysis.*

**Reply**: We have redone Figure S1. It now shows continuous temperature and salinity measurements through the water column. It also shows data from all three stations we scanned shells from (which have identical properties).

The individual measurements of the environmental parameters in Table S1 and S2 now include, as you suggested measurements from the min, mid and max depth. I would also like to reiterate that this water chemistry data is already published online <https://doi.org/10.21335/NMDC‐225800978>.

*Table S1 for station 1110 are the sample numbers correct? You have mean calcite volume data for nets with 0 samples (I am guessing that column E, F, G may not be correct)*

**Reply**: Table S1 has been check and this mistake has been corrected.

*Table (limacine helicina tab in supplement): rearrange rows 31-58 (i.e, the samples with less than or equal and greater than) so they correspond to the value of 2.3 in the text. Explain what Top 5 to 7 correspond with.*

**Reply**: The rows have been rearranged to correspond with the text. The split was made at 2.25 whorls. The measurements for Top 5-7 have been removed because this data was not used in any analysis. Only the measurements from Top 1-4 were used in the analysis, because we could do these measurements on small shells as well.

*Supplementary table. It’s a bit of work, but it might be prudent to include a readme section to explain some of the variables in your table(s).*

**Reply**: This is a great suggestion. A summary of the tables and dataset tabs in the supplementary information file has now been added, the tab is called “ReadMe”.

**References**

1. Brown, S. J. and Elderfield, H.: Variations in Mg/Ca and Sr/Ca ratios of planktonic foraminifera caused by postdepositional dissolution: Evidence of shallow Mg-dependent dissolution, Paleoceanography, 11(5), 543–551, doi:10.1029/96PA01491, 1996.
2. Hecht, A. D., Eslinger, E. V and Garmon, L. B.: Experimental studies on the dissolution of planktonic foraminifera, in Dissolution of Deep-sea Carbonates, vol. 13, edited by W. V Sliter, A. W. H. Bé, and W. H. Berger, pp. 59–69, Cushman Foundation for Foraminiferal Research., 1975.
3. Honjo, S. and Manganini, S. J.: Annual biogenic particle fluxes to the interior of the North Atlantic Ocean; studied at 34°N 21°W and 48°N 21°W, Deep Sea Res. Part II Top. Stud. Oceanogr., 40(1), 587–607, doi:https://doi.org/10.1016/0967-0645(93)90034-K, 1993.
4. Ofstad, S., Meilland, J., Zamelczyk, K. and Chierici, M.: Development, Productivity, and Seasonality of Living Planktonic Foraminiferal Faunas and Limacina helicina in an Area of Intense Methane Seepage in the Barents Sea, J. Geophys. Res. Biogeosciences, 125(2), 1–24, doi:10.1029/2019JG005387, 2020.
5. Schiebel, R. and Hemleben, C.: Planktic Foraminifers in the Modern Ocean, Springer-Verlag, Berlin Heidelberg., 2017.
6. Takahashi, K. and Be, A. W. H.: Planktonic foraminifera: factors controlling sinking speeds, Deep Sea Res. Part A, Oceanogr. Res. Pap., 31(12), 1477–1500, doi:10.1016/0198-0149(84)90083-9, 1984.
