# Supplementary material for: Shell density of planktonic foraminifera and pteropod species Limacina helicina in the Barents Sea: Relation to ontogeny and water chemistry
Source: PLoS One. 2021 Apr 28;16(4):e0249178. doi: 10.1371/journal.pone.0249178 (PMC8081242; doi:10.1371/journal.pone.0249178)
Supplement: Attachment — Submitted filename: Response to Reviewers.docx [file pone.0249178.s010.docx]

Dear Academic Editor,

Thank you for your comments and suggestions for further improvement of the manuscript. We have corrected our manuscript following your annotations in the PDF-file and address each of your four separate comments below.

Yours sincerely,

Siri Ofstad, Katarzyna Zamelczyk, Katsunori Kimoto, Melissa Chierici, Agneta Fransson and Tine L. Rasmussen

*- please introduce the concepts of migration (diel or ontogenetic) much earlier in the text, preferably in the introduction. They are both important for the interpretation of the results. Please also be more careful with the wording since it remains difficult to actually prove ontogenetic migration in foraminifera, it could also be that shells simply grow bigger/denser at a preferred depth.*

**Reply:** Thank you for your comment, these concepts are central to the manuscript and therefore warrants a more in-depth explanation. We have now included the concepts of diel and ontogenetic migration in the introduction and have attempted to make the reader aware that ontogenetic migration is a disputed concept, but we are assuming that it occurs.

*- please explain better what is meant with secondary calcification and how it differs (or not) from encrustation.*

**Reply:** Thank you for bringing this to our attention. Encrustation and secondary calcification are often used interchangeably in the literature, leading to some uncertainty (e.g. Kohfeld et al., 1996; Hillaire-Marcel et al., 2004). We have clarified what we mean by these terms (secondary calcification, gametogenic calcite addition and diagenetic encrustation) in the introduction, so it is clear to the reader what they refer to in this manuscript. We have also corrected instances where we have mixed the terms.

*- please explain earlier and in greater depth how habitat depth and (the pattern in) shell density could be related.*

**Reply:** We have added this to the introduction following the introduction to diel and ontogenetic migration, and clearly state that we hypothesize that depth habitat and shell density are related, if we assume that calcification is continuous and ontogenetic vertical migration occurs. We have also added that we assume that denser shells found below lighter shells are older, as you pointed out in your annotation, we make this assumption later in the results and discussion.

*- the colour plots in figures 3, 4 and 8 are confusing because they suggest three dimensions (variability in the x direction), whereas I think they show the data from a single CTD profile.*

**Reply:** We wholeheartedly agree with this comment and have removed the aragonite and calcite background fill in Figures 3, 4 and 8. You are correct, the aragonite and calcite data are from a single CTD profile, therefore the profiles are now shown as an x and y plot.
